# Efficacy and Safety Profile of Biosimilar Polyethylene Glycol (PEG)-Asparaginase (Asviia) in Patients With Acute Leukemia: A Retrospective Study From Kashmir

**DOI:** 10.7759/cureus.73727

**Published:** 2024-11-15

**Authors:** Faisal R Guru, Rukhsana Akhter, Shumail Bashir, Syed Ahmed Nisar, Mohmad Hussain Mir, Zafirah Zahir, Ulfat Ara Wani, Suyash Bharat, Richa Tripathi

**Affiliations:** 1 Department of Medical Oncology, Sher-i-Kashmir Institute of Medical Sciences, Soura, IND; 2 Department of Pathology, Sher-i-Kashmir Institute of Medical Sciences, Soura, IND; 3 Department of Chest Medicine, Government Medical College (GMC) Baramulla, Baramulla, IND; 4 Department Of Medical Oncology, Sher-i-Kashmir Institute of Medical Sciences, Soura, IND; 5 Medical Affairs, Zydus Lifesciences LTD, Ahmedabad, IND

**Keywords:** acute lymphoblastic leukemia (all), biosimilar peg-asparaginase, hematological parameters, minimal residual disease (mrd), pediatric oncology

## Abstract

Background: Biosimilar pegylated L-asparaginase offers a promising alternative to the innovator molecule for treating acute lymphoblastic leukemia (ALL) in Indian children. It addresses challenges associated with drug availability and cost while providing similar therapeutic advantages. This biosimilar ensures wider access to essential treatment in resource-limited settings such as India.

Materials and methods: A retrospective study was conducted at the Pediatric Oncology unit of the Department of Medical Oncology, Sher-I-Kashmir Institute of Medical Sciences (SKIMS) Srinagar. The study evaluated the efficacy and safety of biosimilar polyethylene glycol-asparaginase (PEG-ASP) (Asviia) in newly diagnosed pediatric ALL patients treated between January 2021 and December 2023. Each patient received two induction doses of PEG-ASP.

Results: The study included 45 patients (29 boys, 16 girls) with a median age of 7.5 years (range: 1-16 years), with most patients diagnosed with Pre-B ALL. The median PEG-ASP dose administered intravenously was 1175 IU (range: 1125-3750 IU). Significant improvements in hemoglobin and platelet counts were observed following the first dose of PEG-ASP. The biosimilar PEG-ASP was well tolerated, with no life-threatening events reported. At the end of the induction phase, 40 patients (88.89%) achieved complete remission with minimal residual disease (MRD) negativity, while five patients had MRD positivity.

Conclusion: The study provides valuable insights into the efficacy and safety of biosimilar PEG-ASP for pediatric ALL in resource-limited settings, with strong data on remission rates and minimal adverse events.

## Introduction

Acute lymphoblastic leukemia (ALL) constitutes nearly 77% of all cancers diagnosed in children under 15 years of age globally [1]. In India, leukemia accounts for approximately 40 to 50% of the total childhood cancer burden, with ALL being the most prevalent type with an incidence rate of up to 101.4 per million in boys and 62.3 per million in girls [2]. Chemotherapy remains the main treatment for pediatric ALL worldwide, with high-income countries achieving a 5-year overall survival rate of around 90% through effective protocols like Children’s Oncology Group (COG) and Berlin-Frankfurt-Münster (BFM), while India shows progress but still lags with survival rates ranging from 45% to 81% due to challenges in uniform treatment, supportive care, and treatment abandonment [3]. Currently, there are three asparaginase preparations available: L-asparaginase (native asparaginase), polyethylene glycol-asparaginase (PEG-ASP) (Asviia) derived from *Escherichia coli*, and *Erwinia* asparaginase sourced from *Erwinia chrysanthemi* [4]. PEG-ASP received FDA approval in February 1994 for intramuscular use in patients with ALL who have hypersensitivity to native forms of L-asparaginase. In November 2005, the FDA approved the intravenous administration of PEG-ASP, and in July 2006, expanded its approval for frontline treatment of ALL patients based on the COG study CCG-1962 [5]. PEG-ASP is the preferred formulation in ALL treatment regimens because of its longer half-life, which extends the circulation time of the enzyme and minimizes immunogenicity compared to native asparaginase [6].

PEG-ASP is a bioengineered compound synthesized through the covalent linkage between *E. coli*-L-asparaginase (EC-ASP) and PEG units. This innovative process, known as PEGylation, involves the modification of biological molecules by the non-toxic and non-immunogenic polymer, PEG [7]. The strategic application of PEGylation allows for the optimization of enzyme activity by facilitating unhindered access to the enzyme’s active sites, while concurrently impeding the uptake of EC-ASP by the reticuloendothelial system [7,8]. PEG-ASP has a longer half-life and lower immunogenicity than unmodified EC-ASP, enabling sustained asparagine depletion with fewer allergic reactions. This allows for less frequent dosing and fewer interruptions in ALL treatment. Additionally, PEG-ASP is more thermally stable and less temperature-dependent, facilitating easier transport and storage, which enhances accessibility in varied clinical settings. This dual mechanism not only enhances the therapeutic efficacy of ASP but also shields its antigenic determinants from immune recognition, thereby reducing the possibility of antibody development [9,10]. Leukemia cells require exogenous asparagine to survive, while normal cells can synthesize asparagine. Asparaginase depletes serum asparagine by converting it to aspartic acid and ammonia. Therefore, this anti-leukemic activity causes deprivation of the critical amino acid, leading to cell death [11].

In addition to its therapeutic benefits, the administration of Asviia in pediatric ALL treatment regimens requires a comprehensive understanding of its associated toxicities. Reported adverse effects include hepatotoxicity, pancreatitis, hypertriglyceridemia, and hypersensitivity reactions, all of which can significantly impact patient outcomes and treatment adherence [12,13]. Recognizing the importance of potential risks associated with PEG-ASP, our study evaluates the efficacy of PEG-ASP and its associated toxicities in pediatric ALL patients within the Indian population.

## Materials and methods

Study objective

The objective of this study was to evaluate the efficacy and safety of the biosimilar PEG-ASP in newly diagnosed pediatric patients with ALL. Primary endpoints included the evaluation of efficacy through improvement in hematological parameters, such as hemoglobin, platelet count, total bilirubin, and serum albumin, following the administration of PEG-ASP. Secondary endpoints included the assessment of the incidence of adverse events, the overall safety profile, and the tolerance of the PEG-ASP among pediatric patients.

Study design and setting

This retrospective, single-center study was conducted at the Pediatric Oncology unit of the Department of Medical Oncology, Sher-I-Kashmir Institute of Medical Sciences (SKIMS) in Srinagar, India.

Participants included pediatric patients aged between 1 and 16 years who were newly diagnosed with ALL and enrolled in the SKIMS pediatric ALL treatment protocol, which incorporates PEG-ASP as part of the induction phase. Patients eligible for this study were those with a confirmed diagnosis of ALL and whose parents or guardians provided consent for participation.

Study participants and treatment protocol

The study included data from 45 pediatric patients diagnosed with ALL between 1st January 2021 and 31st December 2023. The treatment consisted of a standard induction chemotherapy regimen specifically designed for pediatric ALL patients, which included two doses of biosimilar PEG-ASP. Each dose was administered intravenously over a one-hour period at a dose of 2500 IU/m², in conjunction with other agents based on the treatment protocol.

Data collection and statistical analysis

Data for this retrospective analysis included patient demographics - age, gender, and diagnosis - alongside baseline and follow-up laboratory parameters, including hemoglobin, platelet count, total bilirubin, and serum albumin levels. These parameters were measured before and after the administration of PEG-ASP. Adverse events, including febrile neutropenia, allergic reactions, and liver function abnormalities, were recorded to assess the safety profile of PEG-ASP. Additionally, efficacy outcomes, specifically complete remission and minimal residual disease (MRD) status, were evaluated after the conclusion of the induction phase. Statistical analysis involved summarizing continuous variables like laboratory values and age as means or medians, while categorical variables, including gender and adverse events, were presented as frequencies and percentages. Efficacy was assessed through improvements in hematological parameters and MRD status at the end of induction therapy.

Ethical considerations

The local institutional review board (Institutional Ethics Committee- Sher-I-Kashmir Institute of Medical Sciences, Srinagar) approved the study (IEC/SKIMS Protocol # 227/2024), which complied with the Declaration of Helsinki regarding research involving humans.

## Results

**Figure 1:**
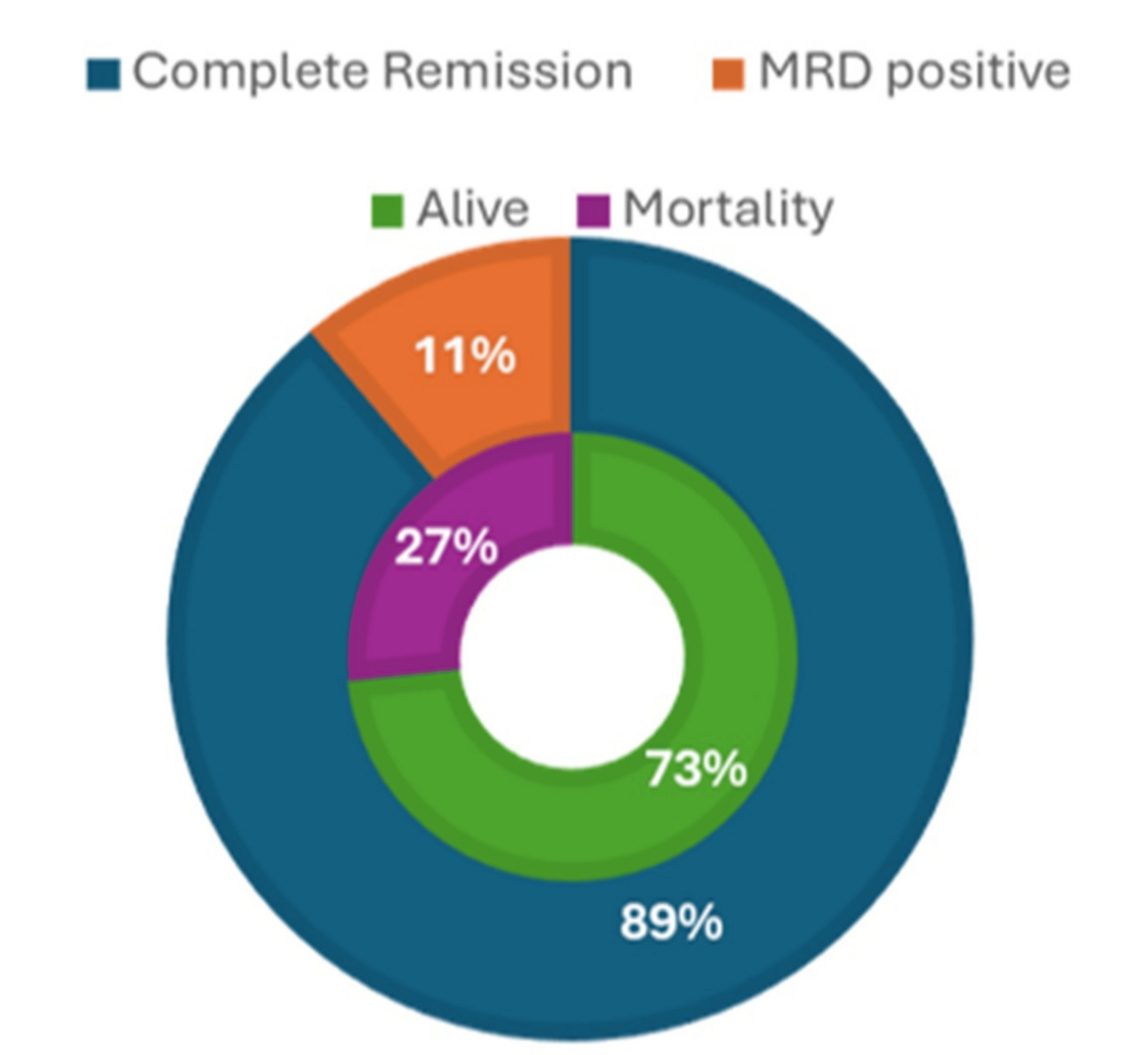
Efficacy data of biosimilar Pegaspargase (Asviia) (N=45) MRD: minimal residual disease

The study population data of 45 patients had a male predominance of 64.5%, with a median age of 7.5 years. The median dose of intravenously administered PEG-ASP was 2500 IU/m². Patient demographics and hematological parameters after PEG-ASP delivery are presented in Table 1. The efficacy data showed that 88.89% of patients achieved complete remission and 11.11% of patients were MRD positive with an overall mortality rate of 26.67% (Figure 1). The study reported 31 adverse events (AE), episodes with sepsis/febrile neutropenia (17.78%) being the most common AEs, followed by vomiting (13.33%), and allergy (13.33%) (Table 2).

**Table 1:** Patient’s demographic and clinical characteristics (n=45) PEG-ASP: peg-asparaginase; IU: international unit

Demography		
Age in years (Median)	7.7	
Gender n (%)	Male	29 (64.5%)
	Female	16 (35.5%)
PEG-ASP dose (Median, IU)	1175	
Laboratory Parameters		
	Prior to first dose of PEG-ASP (mean + SD)	Prior to second dose of PEG-ASP (Mean + SD)
Hemoglobin (g/dL)	7.64 + 2.66	9.03 + 2.35
Platelet /µl	83673 + 94911	114075 + 149571
Total bilirubin (mg/dL)	0.54 + 0.32	1.68 + 5.97
Serum albumin (g/dL)	4.09 + 0.89	3.8 + 0.59

**Table 2:** Safety data of biosimilar Pegaspargase (Asviia)

Adverse Events	Number (n=45)	(%)
Sepsis/febrile neutropenia	8	17.8
Allergy	6	13.3
Vomiting	6	13.3
Hyperglycemia	5	11.1
Transaminitis event	2	4.44
Pancreatitis	2	4.44
Bilirubin >3 mg/dL	2	4.44
Seizure	0	0
Encephalopathy syndrome	0	0
Albumin <2 g/dL	0	0
Life threat/intervention needed	0	0

## Discussion

The efficacy of biosimilar PEG-ASP in treating pediatric ALL is evidenced by an 88.89% complete remission rate, accompanied by significant improvements in hematological parameters, including hemoglobin and platelet counts, showcasing its therapeutic effectiveness. The findings align well with established data on Oncaspar, highlighting the biosimilar’s comparable efficacy and safety profile [14,15]. The referenced pilot study by Venkatagiri et al. (2024) supports PEG-ASP’s therapeutic stability, demonstrating sustained serum asparaginase activity and an absence of significant hypersensitivity reactions or toxicities. This reinforces PEG-ASP’s potential as a cost-effective and reliable alternative in pediatric ALL treatment, with practical relevance for resource-limited settings [16].

The presence of MRD is a critical prognostic factor in ALL. This study highlights that early administration of PEG-ASP significantly accelerates MRD clearance. These findings are supported by the study of Popov et al. (2023), which demonstrated that early administration of PEG-ASP on day 3 of induction therapy improved MRD clearance and long-term outcomes in children with ALL [17]. However, patients who remain MRD-positive after treatment face a less favorable prognosis. Slow MRD responders (≥1% on day 15) generally have poorer outcomes compared to those with rapid MRD clearance. Despite this, PEG-ASP has been shown to improve overall outcomes even in slow responders, particularly in intermediate-risk (IR) patients, leading to better event-free survival (EFS) and overall survival (OS) rates [18].

The reported overall mortality rate of 26.67% in the present cohort shows the significant challenges in managing pediatric ALL patients. Sepsis and febrile neutropenia are common and severe complications in immunocompromised patients, often leading to high mortality rates if not promptly and effectively managed. Additionally, hyperglycemia, often exacerbated by steroid use in ALL treatment, can result in metabolic imbalances that further weaken a patient’s immune response, making them more susceptible to infections and other complications [19,20]. However, the mortality observed in our study could be attributed to the cumulative impact of multiple disease and treatment-related factors.

All patients in the study received PEG-ASP intravenously, a practice influenced by the FDA’s approval of IV PEG-ASP in 2005. The primary difference between administration routes lies in the duration of asparagine depletion; intravenous administration of PEG-ASP, with a half-life of 5.73 ± 3.24 days, sustains asparagine depletion significantly longer compared to the intramuscular administration of native EC-ASP, which has a shorter half-life of 1.28 ± 0.3 days, leading to quicker clearance and shorter depletion duration [21]. The study reported a higher incidence of allergic reactions, possibly due to the intravenous route, which results in prolonged asparagine depletion and an increased likelihood of AE. This observation is supported by studies from Abbott et al. (2015) and Hasan et al. (2017) [22-24].

The study noted an increase in total bilirubin levels and a decrease in serum albumin levels, indicating potential liver toxicity associated with PEG-ASP. Elevated liver enzymes and bilirubin levels, along with reduced albumin levels, suggest liver injury or inflammation, consistent with findings from other studies [17,25]. Therefore continuous monitoring of liver function tests is essential; Tölle et al. (2024) reported that hepatological parameters can be managed through the use of ursodeoxycholate and plasmapheresis [26].

The safety profile of biosimilar PEG-ASP in our study falls within acceptable benefit-risk parameters. Asviia enhances water solubility, improves mobility in solution, increases distribution, decreases immunogenicity, and reduces renal clearance [27,28]. These characteristics contribute to prolonged efficacy and a lower incidence of silent antibody formation. Asviia facilitates the rapid clearance of lymphoblasts-63% by day 7 and 96% by day 14 and maintains sustained asparaginase activity. The major advantages of Asviia include its 2-3-week duration of action and the flexibility of both intravenous and intramuscular administration, enhancing convenience for patients and healthcare providers [29]. Despite these promising findings, the study’s retrospective design and single-center setting may limit the generalizability of the results. The relatively small sample size restricts the ability to detect rare AE, and the lack of stringent inclusion criteria might introduce variability in patient characteristics. Further studies with larger, multi-center cohorts and prospective designs are necessary to validate these findings and provide more comprehensive insights into the long-term safety and efficacy of biosimilar PEG-ASP.

## Conclusions

This study highlights that biosimilar PEG-ASP is an effective and safe option for treating pediatric ALL, achieving nearly 90% complete remission with MRD negativity. The manageable safety profile, with primarily mild and expected AE, highlights its suitability in clinical practice. Importantly, PEG-ASP provides a cost-effective alternative to the innovator drug, making essential leukemia treatment more accessible in resource-limited settings like India. The results show that biosimilar PEG-ASP offers comparable efficacy and addresses key challenges of affordability and availability, which are crucial for improving pediatric ALL treatment outcomes and expanding access to life-saving care for children in need. To improve PEG-ASP’s accessibility in resource-limited settings beyond India, initiatives such as local production to reduce cost, collaboration with WHO for essential drug listing, and government-backed financial support in healthcare programs can enhance affordability and distribution, potentially improving outcomes in underserved populations.
